# Pulsed current-voltage electrodeposition of stoichiometric Bi_2_Te_3_ nanowires and their crystallographic characterization by transmission electron backscatter diffraction

**DOI:** 10.1080/14686996.2019.1671778

**Published:** 2019-09-25

**Authors:** Cristina V. Manzano, Mikhail N. Polyakov, Jon Maiz, Myriam H. Aguirre, Xavier Maeder, Marisol Martín-González

**Affiliations:** a Instituto de Micro y Nanotecnología, IMN-CNM, CSIC (CEI UAM+CSIC) Isaac Newton, 8, E-28760, Tres Cantos, Spain; b Laboratory for Mechanics of Materials and Nanostructures, Empa, Swiss Federal Laboratories for Materials Science and Technology, Thun, Switzerland; c Laboratory of Advanced Microscopy and Department of Physics Condensed Matter, University of Zaragoza, Zaragoza, Spain; d Institute of Nanoscience of Aragón-ICMA-CSIC, University of Zaragoza, Zaragoza, Spain

**Keywords:** Pulsed electrodeposition, bismuth telluride, thermoelectric materials, nanowires, transmission electron backscatter diffraction (t-EBSD), 102 Porous / Nanoporous / Nanostructured materials, 300 Processing / Synthesis and Recycling, 301 Chemical syntheses / processing, 302 Crystallization / Heat treatment / Crystal growth, 503 TEM, STEM, SEM, 504 X-ray / Neutron diffraction and scattering

## Abstract

Bi_2_Te_3_ nanowires with diameters ranging from 25 to 270 nm, ultra-high aspect ratio, and uniform growth front were fabricated by electrodeposition, pulsing between zero current density during the *off* time and constant potential during the *on* time (pulsed-current-voltage method, p-IV). The use of zero current density during the *off* time is to ensure no electrodeposition is carried out and the system is totally relaxed. By this procedure, stoichiometric nanowires oriented perpendicular to the *c-axis* is obtained for the different diameters of porous alumina templates. In addition, the samples show a uniform growth front with ultra-high aspect ratio single crystal nanowires. The high degree of crystallinity was verified by transmission electron backscatter diffraction. This characterization revealed that the nanowires present both large single crystalline areas and areas with alternating twin configurations.

## Graphical Abstract

**Figure:**
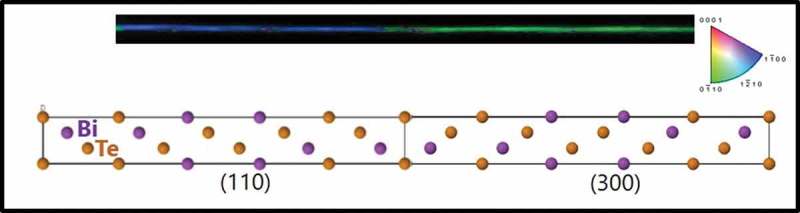

## 1. Introduction

Bismuth telluride is an attractive semiconductor whose principal application is as a thermoelectric material around room temperature. Moreover, Bi_2_Te_3_ belongs to a novel class of quantum materials called three-dimensional topological insulators (3D-TIs) [1,2]. This quantum form of matter presents unique and topologically protected surface states [3–6]. Therefore, in order to study these effects and the possible relations between them, it is important to synthesize Bi_2_Te_3_ nanowires of high crystallographic quality. The thermoelectric figure of merit of nanowires can be increased by lattice thermal conductivity [7,8]. The reduction of this parameter is achieved by the reduction of the size of the nanostructure due to the increment of the phonon scattering, through the reduction of the diameter in the case of the nanowires.

Bi_2_Te_3_ has a rhombohedral structure with space group **D_3_**_*d*_^**5**^ ($R\overset{ˉ}{3}m$), and it can also be described in hexagonal coordinates. In a hexagonal description, the system has a layered structure with five atomic layers as a basic unit (cell), named a quintuple layer (QL). The inter-layer bonding within the QLs is strong because of the dominant covalent character, but the bonding between the QLs is much weaker due to an van der Walls-type interaction. In fact, 3 QLs form the hexagonal supercell containing 15 atomic layers stacking along the *c-axis*; the hexagonal lattice parameters are *a* = 4.383 *Å* and *c* = 30.487* Å*.

Electrodeposition is one of the most important techniques for obtaining nanowire arrays of different diameters [9]. According to the literature, in order to obtain Bi_2_Te_3_ nanowire arrays which display high nucleation and uniform growth, the electrodeposition temperature [10] should be decreased to room temperature and pulsing between two potentials [11] should be used to improve the crystallographic quality of the nanowires. The orientations of Bi_2_Te_3_ nanowires grown in anodic aluminum oxide (AAO) templates by electrodeposition are presented in Table 1. In all the previous work the nanowires were grown by pulsed-voltage electrodeposition, where the electrode is pulsed between two potentials [11–15]. During a pulse, the total current supplied (I_t_) to the electrode is convoluted in two parts: a) capacitive current (I_c_), which is used in changing the double layer, and b) a Faradaic current (I_F_), which is associated with the metal deposition rate. Normally, when pulsing between two voltages, the double layer is not completely discharged and I_F_ never goes to 0.

**Table 1.** A summary of the potentials used and the main crystallographic and compositional characteristics of the nanowires obtained in previous studies.

Potentiostatic deposition mode V*on*/V*off*	Stoichiometry	Measured orientation along the axis direction	TEM image notes	Reference
+60 mV/0V	Bi_2_Te_3_	XRD:(015), (101), (110), and (300) TEM: [110]	Crystallite sizes: 10–70 nm	[12]
−0.7 V/0 V	Bi_1.55_Te_3.45_	XRD: (015) and (110)	Polycrystalline	[13]
−200 mV/ +80 mV	Bi_1.9_Te_3.1_	XRD: [110], [210] TEM: single crystal along basal plane	Crystallite sizes: ~2 µm	[14]
−200 mV/ +80 mV	Bi_1.95_Te_3.05_	TEM: [110], high crystalline quality	15° ± 5° orientation variation	[15]

In this study, electrodeposition is carried out by pulsing the cathode between a reduction potential during the *on* time and zero current density during the *off* time (pulsed-current-potential electrodeposition (p-IV). During the electrodeposition (*on* time), the metal ion concentration decreases at the interface between the substrate and the electrolyte. The function of the zero current density periods is to introduce periods when these ions can redistribute at the interface. During the *off time*, the system is completely relaxed under open circuit potential (OCP), and thus I_F_ is reduced to 0. Since the sample is forced to completely discharge the double layer during the *off* time, it is assured that no electrochemical process is taking place, and the electrodeposit is in an optimal state to allow recrystallization (also known as electrochemical annealing). By changing the types of pulses and allowing the system to relax, we are influencing: a) the electrical double layer at the electrode-electrolyte interface, b) the mass transport, and c) the Bi_2_Te_3_ crystallization. This process influences the crystallographic quality of the deposit and the properties of the semiconducting material obtained. This process has been studied before in our group for films [16], 1D nanowire arrays [6,17,18] and 3D Bi_2_Te_3_ networks [19].

Deposition conditions from the literature are listed in Table 1, and the preferred wire axis orientations are reported [12–15], based on X-ray diffraction (XRD) and transmission electron microscopy (TEM) measurements. In some of these studies, single crystal Bi_2_Te_3_ nanowires were reported. However, some TEM images show smaller crystallite sizes (tens of nanometers) [12,15,20]. To confirm whether the nanowires are single crystalline, many areas along the length of a nanowire must be imaged by TEM, and slight twisting or bending of the nanowire can complicate the TEM imaging. Therefore, confirming single-crystallinity in large areas by TEM is tricky, very time-consuming and can draw to a misleading conclusion in the case of very long nanowires. Other techniques should be used to combine and confirm the nanowire crystallinity over large distances.

Transmission electron backscatter diffraction (t-EBSD or TKD for transmission Kikuchi diffraction) allows for sub-10 nm resolution crystal orientation mapping over micrometer scales inside a scanning electron microscope (SEM) [21,22]. It is, therefore, an appropriate technique for investigating the crystallinity, crystal structure and growth orientation of the nanowires over large areas, resulting in the ability to probe a large number of wires and the entire lengths of the wires. In this work, we study high-quality bismuth telluride nanowires by this technique.

Performing an in-depth study on the crystal quality of the nanowires and their orientations is important for understanding the final thermoelectric properties of the nanowires. Due to the anisotropy of the Bi_2_Te_3_, the electrical and thermal conductivities in the directions perpendicular to the *c-axis* are higher than the thermal and electrical conductivities along the *c-axis* [23–27]. The anisotropy of electrodeposited bismuth telluride films was discussed in previous studies performed by several reports [28,29].

In this work, Bi_2_Te_3_ nanowires with different diameters (25, 60, 70, and 270 nm) were fabricated at 4°C by alternating periods of pulsed electrodeposition reduction potential and zero current density. This method enables the fabrication of oriented Bi_2_Te_3_ nanowires with a high aspect ratio, uniform stoichiometry, and large single crystal areas. The nanowires are studied by XRD, TEM and t-EBSD measurements.

## 2. Experimental details

Bismuth telluride nanowires with different diameters were fabricated by electrodeposition. Commercial templates (Whatman Inc., United Kingdom) were used for nanowires with a diameter of approximately 270 nm. However, homemade AAO templates were used for the other diameters. AAO templates with a pore diameter of 60 nm and 40 nm were grown by a two-step anodization process in 0.3 M oxalic acid under 40 V at 3°C. AAO templates with pore diameters of 15 nm were made by a two-step anodization process in 10 wt. % sulfuric acid and 50 wt. % ethylene glycol as described previously [30,31]. After the anodization, the Al foil was removed in an aqueous solution of CuCl_2_ and HCl, and the barrier layer was opened by chemical etching in 10 wt. % H_3_PO_4_ at 30°C. A layer of 5 nm of Cr and 150 nm of Au was sputtered above the AAO in order to obtain the electrical contact necessary for the electrodeposition process.

A conventional three vertical electrode cell and a potentiostat-galvanostat (Eco Chemie, Model AUT302.0, Metrohm, Netherlands) were used to perform the electrodeposition. Pt mesh, Ag/AgCl electrode, and 150 nm Au (111)/5 nm Cr/AAO templates were used as a counter electrode, reference electrode, and working electrode, respectively. The aqueous solution used was 0.75 · 10^−2^ M Bi^3+^, 1 · 10^−2^ M HTeO^2+^ and 1 M HNO_3_ [32]. The solution was prepared from Sigma Aldrich® bismuth pieces (99.999%), Sigma Aldrich® (99.99%) tellurium powder, and Panreac® 65% nitric acid. In order to obtain nanowires oriented along the [1 1 0] direction, the reduction potential was varied following a method similar to that used by Manzano et al. [16,33]for films. To obtain uniform growth and high aspect ratios in the nanowires, the pulse conditions were studied. The electrodeposition was performed at a constant potential during the *on* time and zero current density during the *off* time. The experiments were carried out at 4ºC.

In order to perform the TEM and t-EBSD characterizations, nanowires were dispersed in the following manner. The porous alumina was dissolved in a phosphoric acid (7 wt. %) and chromic oxide (1.8 wt.%) solution at 45ºC for two days. This solution was filtered with ethanol under vacuum conditions to pick up the nanowires in a similar way to ref [34]. Finally, the filter was immersed in a small flask with ethanol in order to have dispersed nanowires in the solution.

The chemical composition was analyzed using S-3000N energy dispersive X-ray (EDX, Hitachi, Japan) setup with 20 kV accelerating voltage. The cross-sectional structures of the nanowires were studied with a Philips (Netherlands) XL305-FEG field emission scanning electron microscope (FE-SEM) with 20 kV accelerating voltage. Structural properties of the nanowires were characterized using X-ray diffraction (XRD); these measurements were performed while the nanowires were still embedded in the alumina templates. The measurements were performed in a Philips (Netherlands) X´Pert four-circle diffractometer system with Cu-Kα radiation. The crystal nanostructure of the nanowires was evaluated from scanning transmission electron microscopy high-angle annular dark files (STEM-HAADF) images using FEI (United States) Tecnai F30 and FEI Titan G2-probe corrected transmission electron microscopes, both operated at 300 kV, and the stoichiometry was determined by *in situ* STEM-EDX analysis. The t-EBSD measurements were performed in a Lyra dual-beam SEM-FIB (focused ion beam) instrument (TESCAN, Czech Republic). The t-EBSD scans were performed with a 30 kV, 5 nA beam, with step sizes of 5–15 nm. For the STEM and t-EBSD measurements, the nanowires were dispersed on carbon grids very carefully in order to avoid bent or twisted nanowires.

## 3. Results and discussion

**Figure 1.**
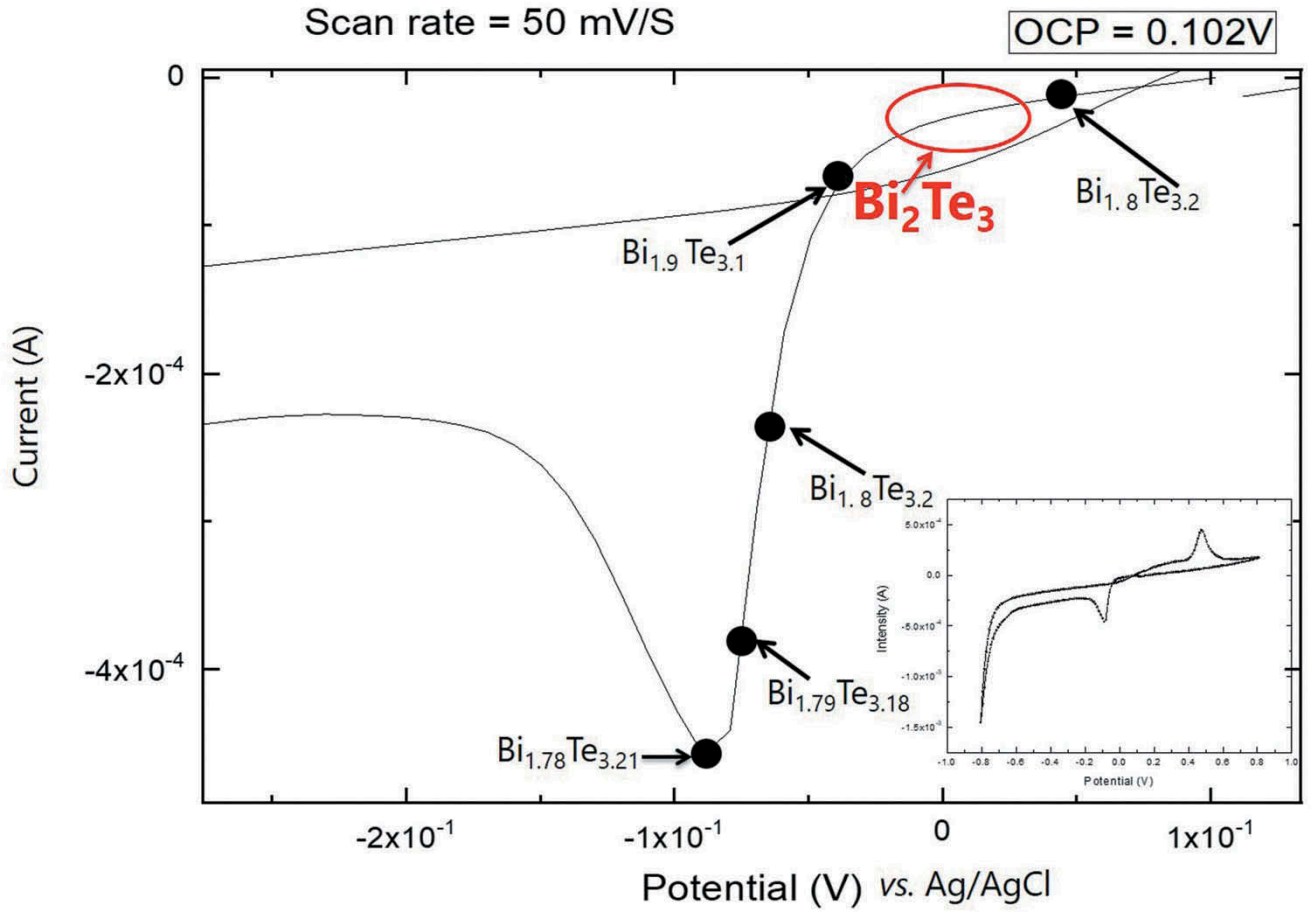
Cyclic voltammetry around the reduction peak of Bi^3+^ (0.75 · 10^−2^ M) and HTeO_2_^+^ (1 · 10^−2^ M) in 1 M HNO_3_. Scan rate 50 mV/s. Inset: cyclic voltammetry from −0.8 V to 0.8 V.

The composition of the nanowires is controlled through the reduction potential. In order to determine the reduction potential necessary to grow nanowires with a composition of 2:3 Bi/Te, cyclic voltammetry was performed. Figure 1 shows the cyclic voltammetry around the reduction peak. In the inset, the cyclic voltammetry from −0.8 V to 0.8 V is shown with a scan rate of 50 mV/s, and the assignment of the different peaks was studied previously in depth [32]. The composition of the nanowires changes around the reduction peak of bismuth telluride. Nanowires with a composition of 2:3 Bi/Te were obtained for the potential inside the ellipse marked in the cyclic voltammetry, at the onset of the bismuth telluride reduction peak. This behavior was observed for all pore diameters studied in this work (25, 60, 70 and 270 nm).

The chemical composition of the nanowires was analyzed by EDX at three different areas along the cross-section of the nanowires. A recent study by Raman spectroscopy of Bi_2_Te_3_ nanowires grown by our group in similar conditions shows a relation between the composition and the Raman spectroscopy, confirming that nanowires with a composition of 2:3 Bi/Te show the three active vibration modes of bismuth telluride without excess Te [17].

In order to improve the nucleation of the nanowires and obtain a uniform growth in the nanowire arrays, the electrodeposition temperature was reduced to 4°C and pulsed current-voltage electrodeposition was applied. The electrode is pulsed alternatively between constant potential (*on* time) and zero current density (*off* time). The applied potential was the potential extracted from the cyclic voltammetry to obtain the Bi:Te ratio of 2:3. The reduction potential was −5, −9, −10 and −50 mV for 25 nm, 60 nm, 70 nm, and 270 nm pore diameters, respectively, and the *off* current was 0 in all the cases. The zero current density was chosen because in this state the system is fully relaxed under the open circuit potential (OCP). The *on* and *off* deposition times were adjusted depending on the pore diameter of the alumina templates to obtain a uniform growth front and the appropriate stoichiometry. The optimization of *on* and *off* times was performed for each diameter in order to obtain a uniform growth in a length of more than 30 µm and the appropriate 2:3 stoichiometry. The *on* and *off* times were 1 s/0.1 s, 1 s/0.1 s, 1 s/0.5 s, and 1 s/0.75 s for 25 nm, 60 nm, 70 nm, and 270 nm, respectively. Since the electrodeposition is performed in an acid solution the final nanowires have a slightly bigger diameter than the initial porous alumina.

**Figure 2.**
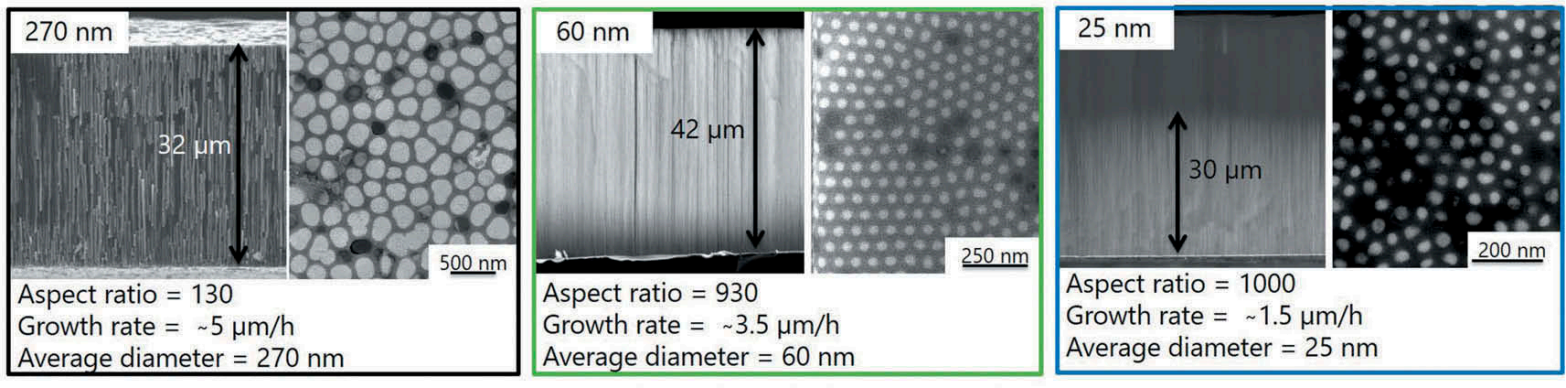
SEM images of some of the Bi_2_Te_3_ nanowires with diameters, 270, 60 and 25 nm grown by pulsed electrodeposition.

Figure 2 shows SEM images of the different nanowire arrays. "In all cases" the nanowires exhibit uniform growth and uniform diameters along the length of the templates. The nanowires are grown perpendicular to the substrate. Uniform growth was observed for a length of 25, 42, 50, and 32 µm for the 25, 60, 70, and 270 nm diameter nanowires, respectively. The aspect ratio (length to diameter) and growth rates are listed in Table 2.

**Table 2.** Growth conditions and aspect ratio of the Bi_2_Te_3_ nanowires prepared by pulsed electrodeposition.

Diameter	Pulse-current-voltage mode V_on_/I_off_	t*_on_*/t*_off_*	Aspect ratio	Growth rate
270 nm	−50 mV/0 mA	1 s/0.75 s	130	5 µm/h
70 nm	−10 mV/0 mA	1 s/0.5 s	715	5 µm/h
60 nm	−9 mV/0 mA	1 s/0.1 s	930	3.5 µm/h
25 nm	−5 mV/0 mA	1 s/0.1 s	1000	1.5 µm/h

**Figure 3.**
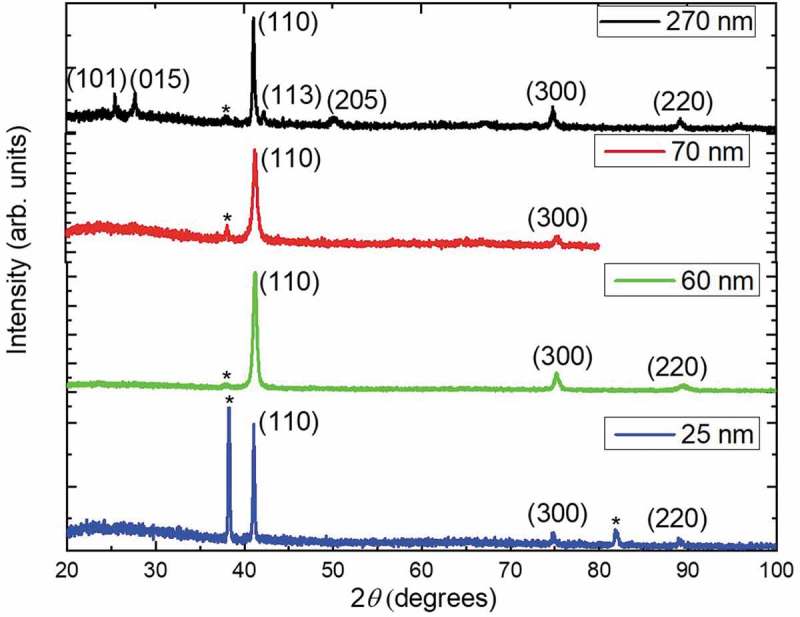
XRD of Bi_2_Te_3_ nanowires with different diameters grown by pulsed electrodeposition. Starred (*) diffraction maxima correspond to the Au substrate.

X-ray diffraction measurements were performed on the nanowires embedded in the alumina templates in order to study the crystallographic orientation of the nanowires (Figure 3). Diffraction maxima associated with the substrate components: Au (JCPDS 04–0784) are observed, as well as those corresponding to Bi_2_Te_3_ (JCPDS 015–0863). For the larger diameter nanowire arrays (270 nm), the detected Bi_2_Te_3_ diffraction maxima are (101) (2*θ *= 23.599), (015) (2*θ *= 27.664), (110) (2*θ *= 41.148º), (113) (2*θ *= 42.153), (205) (2*θ* = 50.315), (300) (2*θ* = 74.955º), and (220) (2*θ* = 89.278º). However, these nanowire arrays appear to be preferentially oriented along the [110] direction. For the smaller diameter nanowire arrays (70, 60, and 25 nm), (110), (300), and (220) diffraction maxima are present, with a strong preferential orientation of the [110] direction parallel to the wire axis, resulting in the *c-axis* being preferentially perpendicular to the wire axis.

To quantitatively determine the degree of preferred orientation of the nanowires, Harris texture analysis [35] was performed. The equation for the texture coefficient is:

(1) $$TC_{\left(hkl\right)\;}=\frac{\frac{I_{\left(hkl\right)\;}\;}{I_{\left(hkl\right)}^{0}}}{\frac{1}{N}\sum \frac{I_{\left(hkl\right)}\;}{I_{\left(hkl\right)}^{0}}}$$

where *I_(hkl)_* and $I_{\left(hkl\right)}^{0}$ are the intensity of a generic diffraction maxima observed in the experimental XRD and the literature value from the database (JCPDS = 015–0863), respectively, and *N* is the number of reflections considered in the analysis. The standard deviation (*σ*) indicates the deviation intensity of the experimental XRD from published values of JCPDS and is calculated as:

(2) $$\sigma =\sqrt{\frac{\sum {\left(TC_{\left(hkl\right)}-1\right)}^{2}}{N}}$$

The values of the texture coefficient and its standard deviation are listed in Table 3. The 270 nm nanowires were grown used a commercial template, and hence their diameter is non-uniform. They exhibit more crystallographic orientations and a wider diameter distribution than samples grown with smaller diameters using home-made AAO templates. The 25, 60, 70 and 270 nm nanowires have higher texture coefficients along (300) than along (110), but since the standard deviation is lower than 1, the nanowires can be considered to be oriented along both crystallographic planes, meaning that they are preferentially oriented along both the [110] and [210] directions, both of which lie in the basal plane of the bismuth telluride structure, perpendicular to the *c-axis* of Bi_2_Te_3_. This analysis demonstrates the importance of determining the texture coefficient of the nanowires because a quick visual inspection of the XRD patterns seems to indicate that the nanowires have a strong preferential orientation only along the [110] direction, while the nanowires in fact also have a preferential orientation along the [210] direction.

**Table 3.** Harris texture coefficient, standard deviation, and diffraction maxima intensity of Bi_2_Te_3_ nanowires grown with different diameters.

Nanowires	Diffraction maxima (*hkl*)	Intensity XRD	Intensity JCPDS	Texture coefficient (*TC_(hkl)_*)	Standard deviation(*σ*)
270 nm	101	12	4	1.52	1.17
	015	12	100	0.06	
	110	36	25	0.72	
	113	6	2	1.58	
	205	5	8	0.30	
	300	7	2	1.81	
70 nm	110	25	25	0.68	0.33
	300	4	2	1.32	
60 nm	110	82	25	0.66	0.34
	300	13	2	1.34	
25 nm	110	396	25	0.73	0.28

**Figure 4.**
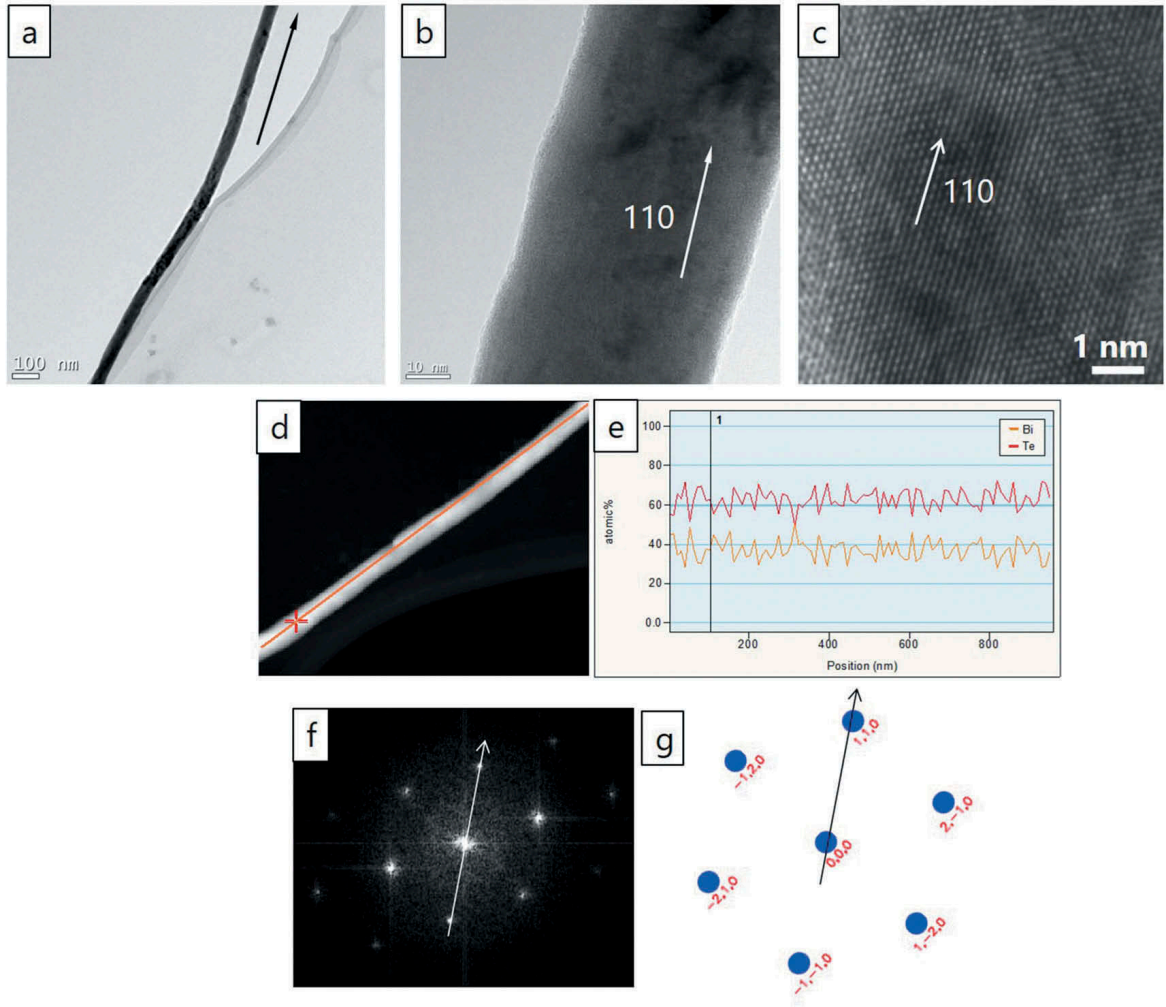
Bright field TEM images of NW with diameter ~60 nm. Different magnification from (a) low magnification to (c) high-resolution TEM showing the direction of growth. (d) Spectrum profile scanning on STEM-HAADF and (e) STEM-EDX profile, showing a constant composition along the length of the wire. (f) and (g) Orientation determined by FFT and description of the indexed diffraction pattern of a typical NW. The direction of growth is maintained along the wire.

These XRD results were corroborated by TEM (Figure 4). Nanowires with diameters from 25 to 270 nm were analyzed by EDX. EDX shows a constant atomic ratio of Bi/Te = 2/3 along the length of microns (Figure 4(d,e)). Nanowires show a [110] direction of growth, which is easily corroborated by high-resolution TEM (Figure 4(c)) and by fast Fourier transform (FFT, Figure 4(f)). The indexed diffraction pattern (Figure 4(g)) also corroborated the [110] orientation as the preferential direction of growth. The area analyses by TEM show long-range crystallinity.

**Figure 5.**
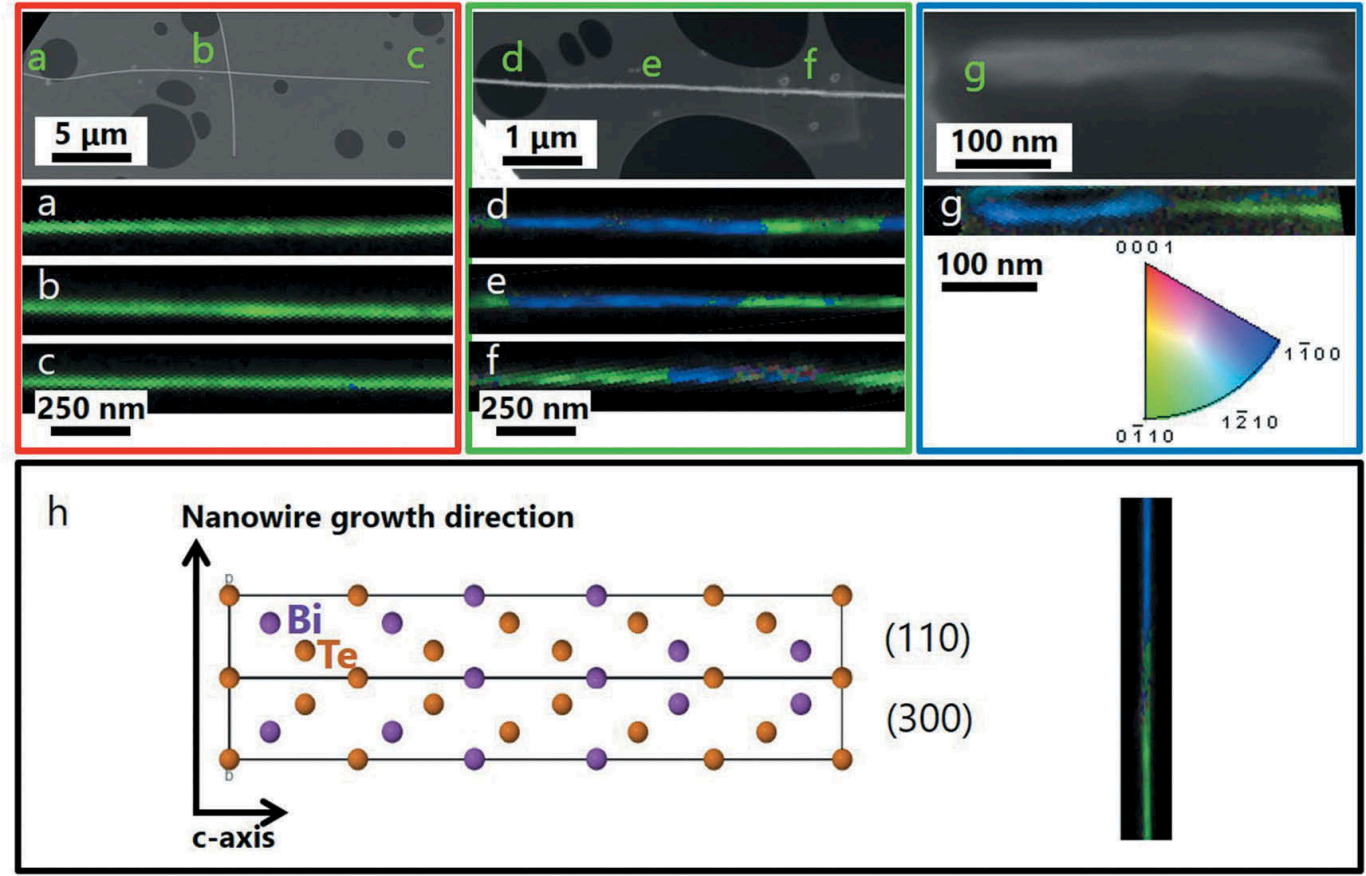
(a–g) SEM images (top) and t-EBSD orientation maps (bottom) are shown for bismuth telluride nanowires with diameters of 70 nm (left), 60 nm (middle), and 25 nm (right) on holey carbon films. The (a–g) labels in the SEM images correspond to the locations of the respective t-EBSD maps. The colors of the orientation maps indicate the orientation along the wire axis direction, with the colors corresponding to the orientation triangle on the right. The wires maintain their crystal orientations along their entire lengths, as evidenced by the wires maintaining their colors in the different regions. There were also some repeating reversals in orientation (e.g. the green/blue/green/blue pattern in t-EBSD map (d) which are characteristic of 60° twin boundaries. (h) Schematic of how the atoms arrange in the observed twins along the nanowires.

In order to confirm that the Bi_2_Te_3_ nanowires maintain their crystal orientation over their entire lengths and to increase statistics exploring several nanowires at the same time, t-EBSD measurements were performed at multiple locations. SEM images and orientation maps are shown in Figure 5, with the locations of the different t-EBSD maps indicated on the overview SEM images. The colors of the t-EBSD orientation maps indicate the orientation of the wire along the wire axis direction, and orientation maps are shown with an image quality overlay (i.e. the dark areas around the wires showed poor or no diffraction patterns, as expected for an amorphous carbon film). The 270 nm wires were too thick and did not produce indexable diffraction patterns. That is why we will only show the results for 70, 60, and 25 nm nanowires.

**Figure 6.**
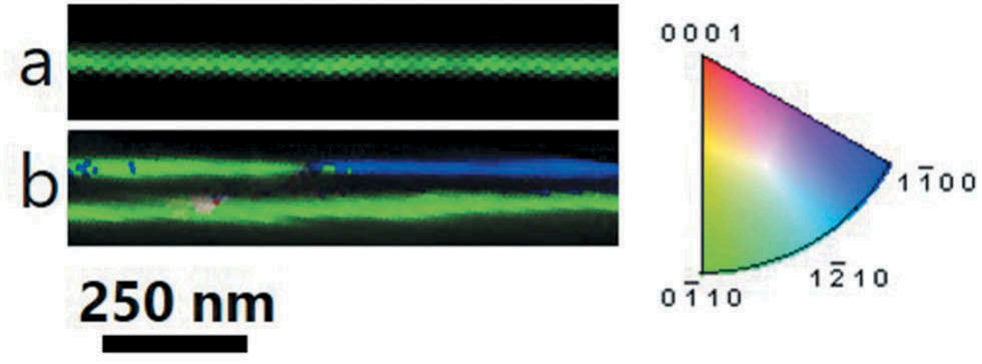
t-EBSD orientation maps are shown for different bismuth telluride nanowires with diameters of 70 nm on holey carbon films. The colors of the orientation maps indicate the orientation along the wire axis direction, with the colors corresponding to the orientation triangle on the right. The areas with the same color indicate the same crystal orientation along the length of the wire. There were also some repeating reversals in orientation (green/blue color reversals in the t-EBSD map, e.g. the top wire in (b)), which are characteristic of twinning. Only a few of the 70 nm nanowires present twinning, while the others are truly single crystals.

For hexagonal and rhombohedral lattice systems, it is possible to use the Bravais-Miller system (with 4 indices) instead of the Miller system (3 indices); for the discussion of EBSD measurement, the Bravais-Miller system is used. In some cases, the 70 nm nanowires (Figure 5 a-c) showed a nearly uniform crystal orientation along their entire length (indicated by a uniform color), with the (0 $\bar{1}$ 1 0) plane perpendicular to the wire axis. This plane, (0 $\bar{1}$ 1 0), corresponds to the [210] direction of bismuth telluride which was measured by XRD, and the (300) plane, and thus confirms those measurements. However, “in other cases” the 70 nm nanowires also showed the 60° twin boundaries [36], which are presented below (Figure 6). The length of the corresponding twins varied between 500 nm and 1.5 µm. Therefore, it can be concluded that, although one nanowire may be single crystalline, not all the nanowires in the same deposition will behave the same. In fact, each of the alumina nanoholes behaves like a nanoelectrochemical cell, and while some of them can produce single crystal nanowires, in other nanoholes, nanowires with defects can be found. This variation must be taken into account. Therefore, it is important to collect enough statistics of the nanowires using TEM or perform t-EBSD in order to obtain a more complete picture of the variety of nanostructures of the nanowires.

The 60 nm nanowires (Figure 5(d–f)) also showed a (0 $\bar{1}$ 1 0) plane perpendicular to the wire axis, but they had many areas in which the orientation twinned back and forth across the (0 $\bar{1}$ 1 0) and (1 $\overset{ˉ}{1}$ 0 0) planes, these planes correspond to [210] and [110] directions of bismuth telluride, respectively. This is illustrated by the green/blue/green sequence along the length of the ‘e’ t-EBSD map. Such twinning has previously been observed in bismuth telluride nanowires [37], and it would not be evident by XRD, because XRD measures only the global structure, without giving an indication of whether the different orientations are occurring within a single nanowire or in separate nanowires. With such twins, a nanowire is not a single crystal, but it consists of two alternating orientations along its entire length, both of which are perpendicular to the *c-axis*. Therefore, the *c-axis* remains perpendicular to the wire axis for both twin orientations. In this case, the length of these twins is between ~300 nm to >1 µm, which gives an aspect ratio from 5 to >16 for the single crystals.

The 25 nm nanowires were difficult to image by t-EBSD, because they were difficult to separate and distribute for imaging, and they would break during t-EBSD sample preparation since they were dispersed ultrasonically. Therefore, the SEM image for the 25 nm sample (Figure 5(g)) actually shows multiple wires stuck together. The t-EBSD map below it is of the wire at the bottom of this agglomeration, and it shows a similar two-orientation twinning configuration as was seen for the 60 nm sample. This was also observed in other 25 nm nanowires. The minimum length of these twins is approximately 200 nm, which gives a minimum aspect ratio of 8.

Therefore, by t-EBSD, the orientations of nanowires were mapped along the lengths of tens of microns. The 70 nm wires were found to be most truly single crystalline, with the wire axis perpendicular to the *c-axis*, but not all of them were single crystalline. The 60 and 25 nm nanowires also had the wire perpendicular to *c-axis*, oriented along the basal plane of the structure, but the wires consisted of alternating twinned regions, rather than a true single crystal. These results are in agreement with the results obtained by XRD and TEM.

In the literature, some studies about bismuth telluride reported that the figure of merit was enhanced by the presence of twinning in films [36] and nanowires [37]. Kwang-Chon Kim and coworkers reported that 60° twin boundaries in bismuth telluride films increase the electrical conductivity by increasing the mobility of the electrons [36]. Moreover, Ho Sun Shin et al. reported that the figure of merit is enhanced by the incorporation of twins into bismuth telluride nanowires because the structure changes the thermal conductivity and carrier concentration. Twins decrease the lattice thermal conductivity by phonon scattering at twin boundaries, due to the reduction of carrier concentration [37].

In our case, we have previously reported a reduction in thermal conductivity from 1.8 ± 0.5 W/K·m for 270 nm to 0.5 ± 0.4 W/K·m for 25 nm nanowires [18]. This reduction is believed to be due to the variation of the mean free path of the acoustic phonons as a result of the nanowire diameter size reduction, as confirmed by the Kinetic–Collective model used to understand such a reduction. Thus, it is not due to the twinning observed in the nanowires. This is indicated by the statistics gathered by the t-EBSD technique, where the smallest observed aspect ratio in our nanowires is at least 5 times the diameter. Such an aspect ratio is enough for the phonons to be scattered preferentially at the nanowire boundaries more than at the twin boundaries. Moreover, we have previously reported an increase in electrical conductivity from (1.0 ± 0.6)·10^4^ S/m for 270 nm to (2.9 ± 0.2)·10^4^ S/m for 50 nm and it was also observed that the surface contribution to total electrical conductivity of the nanowires is 43%–77%, increasing with decreasing diameter, demonstrating the significance of the topological insulator surface states in room temperature NW devices [6].

## 4. Conclusions

Bi_2_Te_3_ nanowires with different diameters ranging from 25 to 270 nm and ultra-high aspect ratio were fabricated by electrodeposition with alternating periods of constant potential during the *on* time and zero current density during the *off* time. Zero current density is used to ensure a true recovery period during the nanowire growth *off* time. With this procedure, stoichiometric nanowires with a 2:3 Bi/Te composition, uniform growth (for a length of 25–50 µm) and ultra-high aspect ratio (10^2^–10^3^) were obtained. The nanowires are mainly single crystalline according to TEM study, with a few inclusions of different nanocrystals. The high degree of crystallinity in these nanowires was verified by t-EBSD. These measurements confirmed the long-range single crystallinity of some nanowires, but in many cases, the nanowires were quasi-single-crystalline, in that they showed alternating twin orientations along their length, with the *c-axis* remaining perpendicular to the wire axis. T-EBSD helps complete the understanding of the structure of the nanowires when combined with SEM and TEM characterizations. Therefore, we can conclude that t-EBSD is a better technique to reach conclusions about the long-range crystallinity in these types of nanowires and to gain statistics.

For nanowires fabricated in this study, the minimum aspect ratio in single crystal region was 5. Therefore, we can confirm that in our nanowires the reduction in thermal conductivity is mainly due to the reduction of nanowire diameter, which produces a variation of the mean free path of the acoustic phonons, and not due to the presence of very small single crystals.
